# Confirmatory Analysis of Nitroimidazoles and Hydroxy Metabolites in Honey by Dispersive-Solid Phase Extraction and Ultra-High Performance Liquid Chromatography-Tandem Mass Spectrometry

**DOI:** 10.3390/molecules23123350

**Published:** 2018-12-18

**Authors:** Xiaowei Li, Yuebin Ke, Yingyu Wang, Chengfei Wang, Dongyang Ye, Xue Hu, Lan Zhou, Xi Xia

**Affiliations:** 1 Beijing Advanced Innovation Center for Food Nutrition and Human Health, College of Veterinary Medicine, China Agricultural University, Beijing 100193, China; xiaowei@cau.edu.cn (X.L.); wangyingyu_1992@163.com (Y.W.); m18811587355@163.com (C.W.); wintersunwyp@163.com (D.Y.); hututuhuhu@163.com (X.H.); m18482100424@163.com (L.Z.); 2 Shenzhen Center for Disease Control and Prevention, Shenzhen 518055, China; keyke@szu.edu.cn

**Keywords:** nitroimidazole, honey, liquid chromatography, mass spectrometry, d-SPE

## Abstract

An ultra-high performance liquid chromatography-tandem quadrupole mass spectrometry (UHPLC-MS/MS) method was developed and validated for confirmatory analysis of four nitroimidazoles and three hydroxy metabolites in honey. Honey samples were dissolved in 2% formic acid solution and nitroimidazoles and metabolites were isolated and enriched by dispersive-solid phase extraction using mixed-mode strong cation-exchange sorbent. The determination involves separation of analytes on an UHPLC C_18_ column and detection by multiple reaction monitoring in positive ionization mode. The recovery of the method was ranged from 90.2 to 105.6% with inter-day relative standard deviations of less than 11.2%. The limits of detection and limits of quantification were in the ranges of 0.02–0.07 µg/kg and 0.05–0.2 µg/kg, respectively. Honey samples from the market were analyzed to demonstrate the applicability of the proposed method.

## 1. Introduction

Metronidazole (MNZ), ronidazole (RNZ), dimetridazole (DMZ), and ipronidazole (IPZ) are 5-nitroimidazole-based veterinary drugs with antibiotic and anticoccidial activities. 5-Nitroimidazoles are primarily used for the treatment and prevention of certain bacterial and protozoal diseases in poultry and swine. They have also been used as feed additives for growth promotion and improvement of feed efficiency. However, they have been banned from use in food producing animals in many countries [1,2] because of their potentially harmful effects on human health [3,4].

Numerous methods have been employed to determine nitroimidazoles and their metabolites in various matrices. Gas chromatography (GC) coupled with nitrogen and phosphorus detection (GC-NPD) [5], high performance liquid chromatography (HPLC) with ultra-violet detection [6] and enzyme-linked immunosorbent assay (ELISA) [7] are less selective for this group of zero tolerance substances. Gas chromatography coupled to negative chemical ionization (NCI) mass spectrometry (MS) is both sensitive and selective for the determination of nitroimidazoles, but derivatisation of non-volatile nitroimidazoles is required prior to analysis [8,9]. Alternatively, LC-MS methods based on thermospray ionization [10], atmospheric pressure chemical ionization (APCI) [11], and electrospray ionization (ESI) [12,13] were developed. More recently, the application of LC hyphenated to tandem MS was reported which combined high specificity, sensitivity and allowed multi-residue determination of nitroimidazoles in complex matrices, together with structural information [14,15,16,17].

In recent years, 5-nitroimidazoles have been used to prevent and control *Nosema apis* in hives [18]. Therefore, sensitive analytical methods for detection of nitroimidazoles and their metabolites including 1-(2-hydroxyethyl)-2-hydroxymethyl-5-nitroimidazole (MNZOH), 2-hydroxymethyl-1-methyl-5-nitroimidazole (HMMNI) and 1-methyl-2-(2′-hydroxyisopropyl)-5-nitroimidazole (IPZOH) are required to control their illegal use in honey production. Cronly et al. [19] developed an LC-MS/MS method for simultaneous determination of chloramphenicol and nitroimidazoles in milk and honey. Honey samples were extracted with acetonitrile and followed by liquid-liquid extraction (LLE) by hexane. For nitroimidazoles, the detection capabilities ranged from 0.66 to 1.98 μg/kg in honey. Guo et al. [20] presented an HPLC-MS/MS method for the determination of nitroimidazoles in honey using molecularly imprinted solid phase extraction. Limits of quantification (LOQ) for different nitroimidazoles could reach 1.0 μg/kg. The aim of this study was to develop a rapid and sensitive method for the identification and quantification of four nitroimidazole and three corresponding metabolites in honey. A sample preparation procedure was evaluated based on dispersive-solid phase extraction (d-SPE) using mixed mode strong cation-exchange sorbent. Instrumental determination was carried out by ultra-high performance liquid chromatography (UHPLC)-tandem MS operating in multiple reaction monitoring (MRM).

## 2. Results and Discussion

### 2.1. Optimization of Sample Preparation

**Figure 1:**
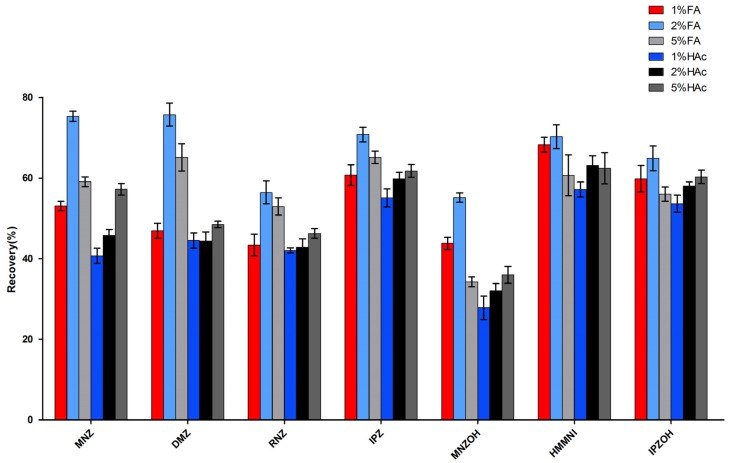
Recoveries of different dissolving solution.

We have demonstrated the applicability of Oasis MCX cartridge which combines strong cation exchange and C_18_ reversed phase interactions in the cleanup and enrichment of nitroimidazoles in swine liver and kidney [17,21]. When testing honey samples in the preliminary experiments, the Oasis MCX cartridge also provided high recovery and clear final extracts, but cartridge clogging which affected the recovery, often occurred with different kinds of honey and was likely due to the complexity of the honey matrix. To address this problem, we tried to apply d-SPE using MCX sorbent which could omit sample loading compared to conventional SPE steps. The dissolving solution of honey sample is critical for the following d-SPE procedure. Since an acidic solution is required for ion exchange adsorption on the MCX sorbent, formic acid (FA) (1%, 2%, 5%) and acetic acid (HAc) (1%, 2%, 5%) solutions were tested for honey dissolution and analytes locking onto the MCX sorbent. As depicted in Figure 1, 2% FA solution yielded the best recoveries for all of target compounds calculated without internal standards. The amounts of MCX sorbent (0.1, 0.2, 0.5 and 1 g) needed for 1 g of honey sample were also evaluated. The results indicated that at least 0.5 g of MCX sorbent should be used to obtain satisfactory recoveries.

### 2.2. Matrix Effect

The recovery of the method may be affected by ion suppression/enhancement due to the presence of undesirable sample components that co-elute with the analytes. Therefore, matrix effects were investigated by comparing the signal intensity of matrix-matched standard solution with that of the standard solution at different concentrations. Slope ratios of the matrix slope versus solvent slope are presented in Table 1 for different kinds of honey. Ion suppression ranged from 23% to 81% was observed for all of the analytes using external standard calibration; meanwhile, the slope ratios using internal standard calibration were between 94% and 101%, which means that use of the isotope-labeled internal standards could effectively compensate for the matrix effect. In this condition, internal standard calibration curves could be used directly for quantification.

### 2.3. Method Validation

**Figure 2:**
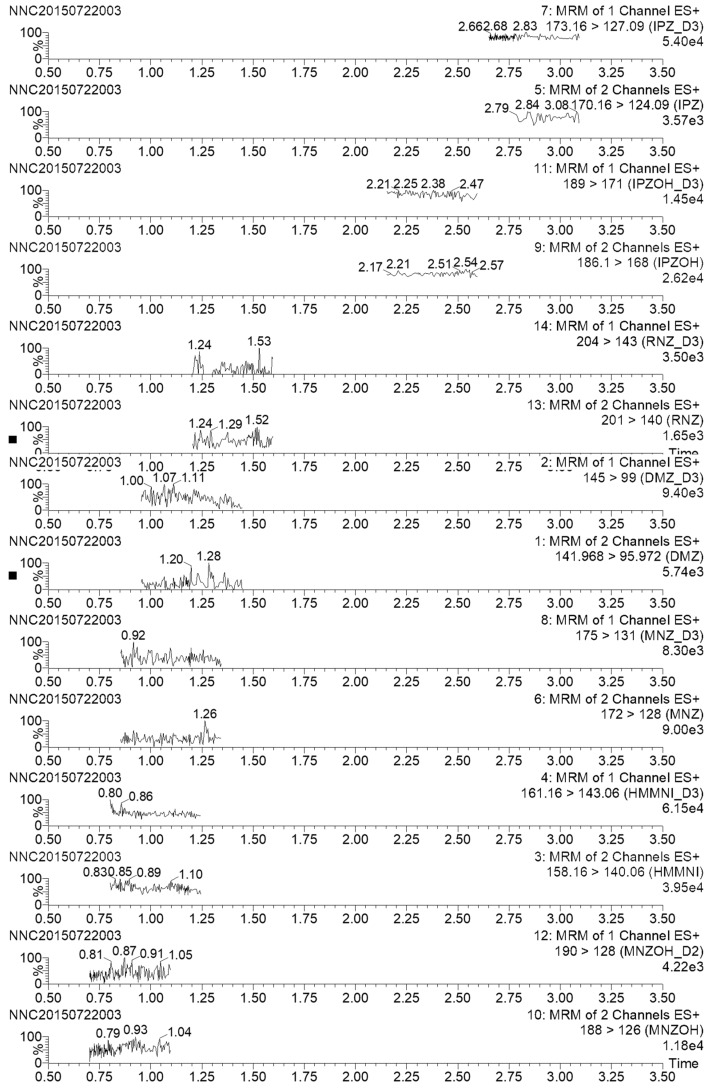
Chromatograms of blank honey samples.

**Figure 3:**
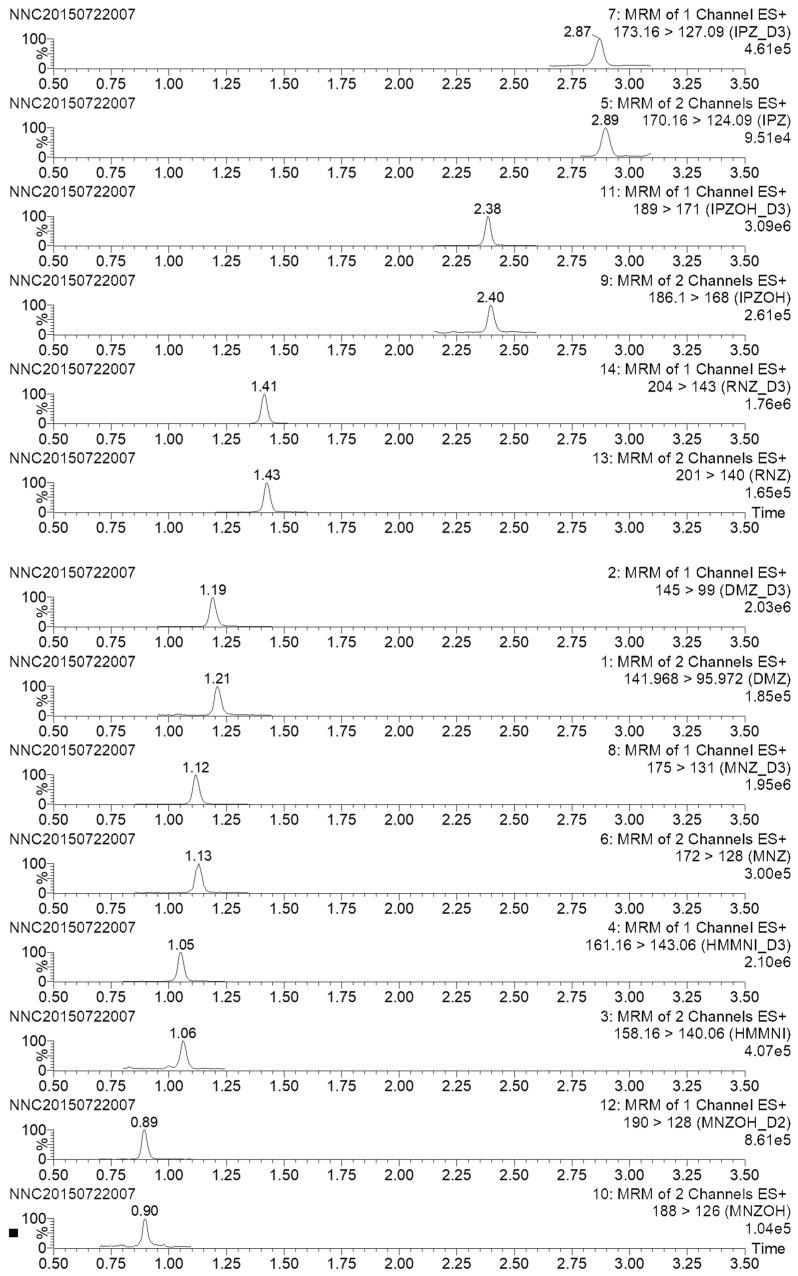
Chromatograms of spiked samples.

The selectivity of the method was investigated by analyzing blank honey samples from different origins. No interfering peak was observed at the retention time of all of nitroimidazoles and internal standards (Figure 2). Over the calibration range (0–500 μg/L), all of the calibration curves presented significant linearity according to coefficient values (r2 ≥ 0.993) with deviations of the individual points from the calibration curves lower than 12%. The results of recovery, and precision, expressed as intra-day and inter-day repeatability, are summarized in Table 2. Nitroimidazoles were fortified in blank sample at the concentrations of 0.2, 0.5, and 1.0 μg/kg in six replicates on three different days, and then extracted and analyzed using the proposed method. The recoveries of nitroimidazoles ranged from 90.2% to 105.6% with inter-day relative standard deviations (RSDs) of <11.2%. The method demonstrated good recovery and precision for quantifying nitroimidazoles in honey samples. Typical chromatograms of fortified honey sample are presented in Figure 3. The limits of detection (LODs) and LOQs were defined as the concentration in the sample that would give a minimum signal-to-noise ratio (S/N) of 3 and 10, respectively, and ranged from 0.02 to 0.07 μg/kg and from 0.05 to 0.2 μg/kg, respectively (Table 2).

### 2.4. Analysis of Real Samples

To examine the feasibility of the proposed method for determining nitroimidazoles in real samples, we analyzed 42 honey samples collected from local markets. MNZ was detected in three samples and the identification of compound was performed by comparing the retention time and ion ratio of real samples to that of the standard. The confirmatory and quantitative results of the contaminated honey samples are summarized in Table 3.

## 3. Materials and Methods

### 3.1. Reagents and Materials

The analytical standards DMZ, RNZ, MNZOH, HMMNI, DMZ-*d*_3_, RNZ-*d*_3_, MNZOH-*d*_2_, and HMMNI-*d*_3_ were obtained from WITEGA (Berlin, Germany). MNZ, IPZ, IPZOH, IPZ-*d*_3_, IPZOH-*d*_3_, and MNZ-*d*_3_ were provided by the EU Reference Laboratory for Residues of Veterinary Drugs (BVL, Berlin, Germany). HPLC grade methanol, acetonitrile, FA, and HAc were purchased from Fisher Scientific Inc. (Pittsburgh, PA, USA). Ammonium hydroxide was provided by Beijing Chemical Co. (Beijing, China). Water was purified using a Milli-Q Synthesis system from Millipore (Bedford, MA, USA). MCX sorbent was purchased from DURLab (Beijing, China). Syringe filters (GHP ACRODISC, 0.2 µm) were purchased from Pall Corporation (Ann Arbor, MI, USA).

Individual stock standard solutions and internal standard solutions were prepared in methanol at a concentration of 1000 mg/L. Mixed working standard solutions were prepared by subsequent dilution with methanol. These solutions were stored at −20°C and were stable for at least 6 months.

### 3.2. Sample Preparation

An aliquot of honey (1 g) was weighed into a centrifuge tube (15 mL), spiked with internal standards at 2.0 μg/kg and equilibrated for 10 min. Honey samples were dissolved in 3 mL of 2% FA solution by vortexing for 1 min. The MCX sorbent (0.5 g) was added and the mixture was vortexed for 2 min. After centrifugation at 10000 rpm for 5 min, the aqueous layer was discarded. Three mL of methanol was then added and the mixture was vortexed for 1 min. The mixture was centrifuged again at 10000 rpm for 5 min, and the upper layer was discarded. The sample was extracted with 3 mL of 5% ammonium hydroxide in methanol by vortexing for 1 min and centrifuged at 10000 rpm for 5 min. The supernatant was collected and the extraction solution was evaporated to dryness in a water bath at 40 °C under nitrogen. The dried residue was reconstituted in 0.5 mL of 0.1% FA-acetonitrile (95:5, *v*/*v*) and filtered prior to UHPLC-MS/MS analysis.

### 3.3. UHPLC-MS/MS Analysis

Chromatographic separation was performed on a Waters Acquity ultra performance liquid chromatography system with column oven temperature maintained at 40 °C, using an Acquity BEH C18 column (50 × 2.1 mm I.D., 1.7 μm particle size; Waters, Milford, MA, USA). 

The injection volume was 10 μL. The mobile phase was composed of 0.1% FA in water (solvent A) and 0.1% FA in acetonitrile (solvent B) at a flow rate of 0.4 mL/min. UHPLC linear gradient conditions were optimized as follows: 0–0.2 min, 95% A; 0.2–2.0 min, 95–60% A; 2.0–2.5 min, 60–0% A; 2.5–2.6 min, 0–95% A; 2.6–4.0 min, 95% A. The MS/MS detection was performed using a Micromass Xevo TQ-S triple quadrupole mass spectrometer (Waters, Manchester, UK). The instrument was set to collect data in MRM mode. Typical interface conditions were optimized for the maximum intensity of the precursor ions as follows: capillary voltage, 2.5 kV; source temperature, 150 °C; desolvation temperature, 500 °C; desolvation gas flow rate, 800 L/h; cone gas flow rate, 150 L/h. Argon was used as collision gas at 0.15 mL/min. The MRM transitions and optimized cone voltages and collision energies are summarized in Table 4.

## 4. Conclusions

In summary, a UHPLC-ESI-MS/MS method has been developed for the determination of nitroimidazoles and metabolites in honey at trace level. Dispersive-solid phase extraction with MCX sorbent served as a simple method to extract and concentrate the target compounds from the matrix for further analysis. The analytes were well separated in 4 min and the method LOQs were at sub microgram per kilogram level. The method was validated and successfully applied to the analysis of contaminated honey samples.

## Figures and Tables

**Table 1:** Slope ratios of matrix-matched standard versus pure standard in solvent.

Analytes	External Standard (%)				Internal Standard (%)			
	Acacia Honey	Jujube Honey	Chaste Honey	Linden Honey	Acacia Honey	Jujube Honey	Chaste Honey	Linden Honey
MNZ	35	23	33	30	96	95	94	96
DMZ	45	41	47	43	96	96	98	99
RNZ	40	47	45	49	98	101	98	96
IPZ	76	80	81	80	97	98	97	98
MNZOH	33	37	35	35	96	96	95	96
HMMNI	54	55	52	60	98	97	99	99
IPZOH	73	70	68	75	98	100	96	96

**Table 2:** Recovery, precision, and sensitivity of the method.

Analyte	Spiking Level (μg/kg)	Recovery (%)	Intra-Day RSD (*n* = 6, %)	Inter-Day RSD (*n* = 18, %)	LOD (μg/kg)	LOQ (μg/kg)
MNZ	0.2	90.8	7.8	8.2	0.02	0.05
	0.5	105.6	5.2	8.9		
	1.0	96.3	4.3	7.3		
DMZ	0.2	101.8	8.8	10.1	0.05	0.1
	0.5	97.8	6.7	8.7		
	1.0	93.4	5.7	7.8		
RNZ	0.2	93.8	9.0	11.2	0.02	0.05
	0.5	98.2	8.6	10.6		
	1.0	97.7	7.9	9.4		
IPZ	0.2	90.2	6.4	8.0	0.05	0.1
	0.5	93.6	6.2	9.9		
	1.0	96.9	5.8	9.3		
MNZOH	0.2	92.3	7.1	10.3	0.07	0.2
	0.5	94.6	7.0	8.7		
	1.0	95.0	4.7	8.5		
HMMNI	0.2	92.6	8.6	9.2	0.07	0.2
	0.5	94.1	9.9	10.5		
	1.0	97.2	7.2	9.5		
IPZOH	0.2	98.3	4.8	6.4	0.07	0.2
	0.5	104.2	6.5	7.8		
	1.0	92.7	5.1	7.9		

**Table 3:** Confirmatory and quantitative analysis of honey samples.

Sample	Analyte Detected	Product Ions (*m*/*z*)	Incurred Sample		Standard		Level (μg/kg)
			RT (min)	Ion Ratio ^a^	RT (min)	Ion Ratio	
Honey 5	MNZ	82/128	1.13	0.5726	1.13	0.5812	0.6
Honey 17	MNZ	82/128	1.13	0.5711			0.3
Honey 19	MNZ	82/128	1.13	0.5738			1.1

^a^ confirmatory transition versus quantitative transition.

**Table 4:** MS/MS transitions and optimal conditions.

Analyte	Precursor Ion (*m*/*z*)	Product Ions (*m*/*z*)	Cone Voltage (V)	Collision Energy (eV)
MNZ	172	82/128 *	15	20/10
MNZ-*d*_3_	175	131	15	10
DMZ	142	81/96 *	10	18/14
DMZ-*d*_3_	145	99	10	14
RNZ	201	55/140 *	20	15/10
RNZ-*d*_3_	204	143	20	10
IPZ	170	109/124 *	32	24/18
IPZ-*d*_3_	173	127	32	18
MNZOH	188	123/126 *	20	15/10
MNZOH-*d*_2_	190	128	20	10
HMMNI	158	55/140 *	18	18/10
HMMNI-*d*_3_	161	143	18	10
IPZOH	186	122/168 *	20	15/10
IPZOH-*d*_3_	189	171	20	10

* Transition for quantification.
